# Key elements and effects of cardiovascular disease management programs based on community-based participatory research: protocol for a scoping review

**DOI:** 10.1186/s13643-021-01804-4

**Published:** 2021-09-24

**Authors:** Juhyeon Yang, Eunsim Kim, Bohyun Park

**Affiliations:** Department of Nursing, Changwon National University, Gyeongnam, 51140 Republic of Korea

**Keywords:** Cardiovascular diseases, Community-based participatory research, Scoping review

## Abstract

**Background:**

Cardiovascular diseases (CVDs) are health problems that demonstrate high death and prevalence rates, and exhibit large health inequalities across different socio-economic status. Although interest in community-based participatory research (CBPR) is increasing because of the efforts to improve health equity, not enough literature review has been conducted on CBPR-based CVD management programs. The objective of this scoping review is to identify the key elements that should be considered when developing CBPR-based CVD management programs, and explore the effects of CBPR-based CVD management programs.

**Methods:**

This study will use the databases of PubMed, Cochrane, and Cumulative Index to Nursing and Allied Health Literature (CINAHL) including grey literature. The criteria for selecting literature will be research that was published in or after 2000, applied CBPR, and either developed or implemented CVD management programs. No limit will be placed on the research design or method. Data extraction will be conducted independently by two researchers, and in the case of data mismatch, a consensus will be reached through discussion. The extracted data will be combined through narrative synthesis.

**Discussion:**

This scoping review will identify specific methods in the development and implementation process of CBPR-based CVD management programs, as well as the characteristics of the programs that were shown to be effective. Therefore, it will be able to provide specific guidelines to researchers, government agencies, and local organizations to design and implement participatory health promotion programs related to CVDs.

**Systematic review registration:**

Open Science Framework 10.17605/OSF.IO/ZW2UY

**Supplementary Information:**

The online version contains supplementary material available at 10.1186/s13643-021-01804-4.

## Background

Cardiovascular diseases (CVDs) are one of the leading causes of death according to the World Health Organization and are believed to be responsible for 18 million deaths worldwide every year [1]. The high CVD morbidity and mortality rates not only add to the socio-economic burden, but can also exacerbate health inequality [2]. Higher CVD death rates in people of lower socio-economic status [3, 4] is a universal phenomenon that occurs in various countries. The reason for the disparities in CVD mortality according to income level is that the factors affecting CVD occurrence or death differ according to income level [3, 5, 6]. In other words, health inequality according to socio-economic status affects the determinants of health and the health status. As reducing this inequality is a goal that does not need a room for debate, various efforts are being made across the world to achieve it. Community-based participatory research (CBPR) is one of the suggested methods.

The CBPR is a community-based participatory research approach that involves a community’s members, leaders, and academic researchers as equal participants [7]. In this manner, CBPR centers on the equality between participants and the fostering of a cooperative partnership for health promotion [8]. It is a method through which members can identify the on-site challenges themselves and produce ideas for solutions for their community that can be implemented more effectively regarding the validity and durability [9]. Through this process, the relevant parties within a community can form and strengthen networks, while improved leadership can lead to heightened autogenous and continuous capacity for better health in the community [10]. While CBPR emphasizes “participation” of the community’s members, it also emphasizes “action” to almost the same degree. In other words, CBPR is a concept that includes action, as well as the process of achieving it through community capacity building and networks [11]. Therefore, CBPR can be used effectively as one of methods to connect academic research findings to on-site health services.

The community capacity is known to be a mediating factor that decreases the gap between the health status and socio-economic status. Health promotion programs based on CBPR are grounded in this mechanism and aim to build community capacity through mediating variables like social networks, support, and social capital, thereby reducing health disparities and improving overall health status. Specifically, they emphasize the importance of addressing social determinants of health, which are defined as factors related to the social environment in which an individual is born, grows up, and lives, to reduce CVD risk factors and health inequalities arising from CVDs [12]. The American Heart Association has even pointed out that “at present, the most significant opportunities for reducing death and disability from CVD in the United States lie with addressing the social determinants of cardiovascular outcomes,” stressing the importance of raising awareness within communities to improve cardiovascular health [13]. Accordingly, it is possible to expect improved results from CBPR-based CVD management programs compared to the non-CBPR-based CVD management programs. As community capacity building cannot be achieved in a short period, considerable time will be required before health status increase. Nonetheless, because a strengthened community capacity does not easily dissolve unless the members’ change, its effects can be expected to be long-term.

Many previous reviews of CBPR in the health and medical sector are focused on specific ethnic groups such as Hispanics, African-Americans, Asian-Americans, and immigrants [14, 15], or residents in specific regions such as Sub-Saharan Africans and Asia-Pacific Islanders [7, 16]. There are not enough reviews that focus on providing a comprehensive compilation of the effects of CBPR-based health programs.

## Methods/design

### Study objectives

The purpose of this scoping review is to identify the key elements that should be considered when developing CBPR-based CVD management programs and to explore the contents and outcome of the CBPR-based CVD management program.

### Protocol design

This scoping review was designed based on the scoping review methodology developed by Arksey and O’Malley [17] and revised by Levac et al. [18]. It composed of 6 components: (1) step 1, identify the research question; (2) stage 2: identifying relevant studies—search strategy; (3) stage 3, study selection; (4) stage 4, charting the data; (5) stage 5, collating, summarizing, and reporting the results; and (6) stage 6: consultation exercise. Preferred Reporting Items for Systematic Reviews and Meta-Analyses for Scoping Reviews [19] will be complied with to report all recommended results.

#### Stage 1: identifying the research questions

The research questions were divided into two streams through a preliminary investigation on the relevant literature. The first is the CBPR-based CVD management program development “process” and the second is about the “outcome” after application of the program. The 'outcome' is subdivided into quantitative and qualitative aspects.

1. What are the key elements that should be considered in the development process of a CBPR-based CVD management program? Focusing on establishing partnerships, applying CBPR principles, and using of field activists
2. What are the contents and outcome of the CBPR-based CVD management program?
  A. How was the program structured and what were the outcomes?
  B. What were evaluated in the qualitative studies and how were the outcomes?

#### Stage 2: identifying relevant studies—search strategy

This study will use the databases of PubMed, Cochrane, and Cumulative Index to Nursing and Allied Health Literature (CINAHL). ProQuest Dissertation and Theses, and Open Grey will be searched for grey literature. We established the search strategies by examining in the abstracts and full texts of previous studies related to this study. The search terms “cardiovascular disease,” “vascul* disease,” and “CVD” for the target population were combined using the Boolean operator “or.” The search terms “community- based participatory research,” “participatory action research,” “CBPR,” “PAR,” “community engagement,” “community involvement,” and “civic engagement,” for the intervention were combined using the Boolean operator “or.” The Boolean operator “and” was used to further combine search results for intervention and target population. For a more comprehensive search, we searched all search terms in Medical Subject Headings, title, and abstract fields. The search strategies were presented in the Additional file 1: Appendix. The search results shared with all researchers of this study and saved in the bibliographic management program EndNote (V.9.3.3.), which will also help in removing duplicate publications.

#### Stage 3: study selection

In order to elicit answers for the research questions of this scoping review, we have determined the selection criteria for this review as shown in Table 1 and will conduct the study selection using those criteria. In general, scoping reviews are used to examine the scope and nature of research activities in the field, and this study also has such reasons. Therefore, various research designs were included in the selection criteria to meet the reasons.

**Table 1:** Inclusion and exclusion criteria

	Inclusion criteria	Exclusion criteria
Publication date	Published in or after 2000	
Study design	Quantitative study (randomized trial, clustered randomized trial, non-randomized trial, repeated measures study, cohort study, case-control study, interrupted time series study, controlled before-after study, before-after study) Qualitative study Mixed method	Quantitative study (non-comparative study) Non-original study (reviews, letters, opinions etc.)
Participants	Adults who are 18 years old or over Community residents	Patients/clients visit or admit to facilities, clinics, or hospitals
Intervention	CVD management program based on CBPR Including at least one among the five components composing the CBPR quality assessment tool developed by Viswanathan et al. [20] and revised by Chen et al. [21]	

The studies retrieved using the search terms presented in the search strategy will be reviewed according to the study selection criteria. Two researchers will review the titles and abstracts of the studies independently and the studies that do not meet the criteria will be excluded under the agreement of the both researchers. If it is difficult to select the literature based on the abstract, the full text of the study will be reviewed to determine its selection. In the case where the two researchers disagree on the selection, the study will be selected after a consensus is reached through sufficient discussion between them. If a consensus cannot be reached, the study will be selected after discussion with a third researcher.

#### Stage 4: charting the data

The two researchers will extract data independently and compare their results. The data will be extracted using the standardized form in Tables 2, 3, and 4. Before starting data extraction, the researchers will compose the data extraction form and attempt the data extraction and, if necessary, will adjust the form. The researchers will meet regularly twice a week for 2 to 3 h to examine and compare the extracted data.

**Table 2:** General characteristics of selected studies

Purpose of study	Development/evaluation of the program
Study design	Quantitative/qualitative/mixed
Demographic characteristics of participants	Race/gender/age
Other characteristics of participants	Health status

**Table 3:** Key element of development of CVD program based on CBPR

Establishing partnership	Composition of community partners
Application of CBPR principle	Planning/execution/topic selection/adaptation/analysis/dissemination
Community activist	Role/training process

**Table 4:** The structure and outcome of the CVD management program based on CBPR (example)

Authors (year)	Study design	Study participants	Intervention			
			Session/duration	Name of intervention “contents in detail”	Method and media	Outcome (statistical significance)
Lynch et al. (2019)	Before-after	age ≥ 18, *N* = 206 (mean age, 57.5)	2 h × 24 times/9 months	Abundant Living in Vibrant Energy (ALIVE) intervention “nutrition, physical activity”	Bible study, small group sessions, church-wide activities, videos, handouts, self-monitor vegetable consumption, bulletin	Vegetable servings consumed/day, total diet quality: (+) Weight, blood pressure: (+)

#### Stage 5: collating, summarizing, and reporting the results

The result of research question 1 — key elements for development of CVD management program based on CBPR — will be extracted using conceptual model that is taken from the CBPR quality assessment tool developed by Viswanathan et al. [20] and revised by Chen et al. [21]. The assessment tool consists of five questions in two domains. The first domain was composed of two items: “community partner identified?” and “community partner involved in the planning and/or execution of research?” The second domain was composed of three items: “community partner involved in selection of research topic or development (or review) of the program?”, “community partner involved in analysis and/or interpretation of research?”, and “community partner involved in dissemination of research results?”

The results of research question 2A — program structure and outcome — will be presented as the program content and measurement before and after the program. To this end, we will review the methods and the results of the selected research. The selected studies are expected to not only use various research designs, but also use several indicators such as health status, knowledge, and satisfaction. Therefore, we will describe pre-post changes to the indicators but not present summarized values. The outcome synthesis of research question 2B — program evaluation through qualitative method — will be described narratively.

The quality of CBPR for the selected studies will be assessed. The quality of CBPR will be assessed using tools developed by Viswanathan et al. [20] and revised by Chen et al. [21], following additional revisions by the researchers of this study. Each question will be measured by a three-point scale (1 = poor, 2 = fair, 3 = good), where a higher score indicates a higher quality of the CBPR. Since various types of experimental study designs are included in the selection criteria of this scoping review, many forms of studies are expected to be selected as a result of the review. Contrary to systematic review, scoping reviews have a wide range of scope, are not specific in terms of research questions, and do not necessarily require quality assessment for risk of bias [17]. Therefore, in this review, quality assessment will not be conducted for the selected studies.

#### Stage 6: consultation exercise

To achieve the objective of this review to identify the key elements of the CBPR-based CVD management program and to assess the effectiveness of the program, we will take consultation from relevant experts on how appropriate the results of the review are. Specifically, we will organize a focus group of researchers or activists who have participated in CBPR, and we will conduct a focus group interview to confirm if the results from this review adequately reflect the needs or experiences of them.

## Discussion

The objective of this scoping review is to examine the literature on CBPR-based CVD management programs, and thereby identify the key elements that should be considered when developing a CBPR-based CVD management program and assess the programs’ effects. This study will be the first scoping review on CBPR-based CVD management programs. As communities are units that not only share geographic boundaries but also identity, it is important to consider the unique environment and context [10]. By examining the development process of CBPR-based CVD programs, this review will be able to identify how a community’s unique character is reflected in the development process. Also, as the health issue that this study is interested in is CVD, this review will identify the content, duration, and resident participation mechanism methods of CBPR-based CVD management programs that have been effective. This study will identify specific methods related to the development and implementation of CBPR-based CVD management programs (e.g., investigating health issues and selecting topics, forming community partnerships, recruiting participants, collecting and analyzing data, utilizing research findings) as well as the characteristics of programs that were shown to be effective. The findings of this study will be able to offer specific guidelines for researchers, government agencies, and community organizations seeking to design and implement health promotion programs using community participation in areas with high CVD prevalence rates.

## Supplementary Information

**Additional file 1:.** Appendix. Search Strategies by the Electronic Database

## Data Availability

The datasets to be generated and analyzed in this study will not be publicly available and will be available by contacting the corresponding author on reasonable request.

## Abbreviations

CBPR: Community-based participatory research
CVD: Cardiovascular disease
