# Supplementary material for: Key elements and effects of cardiovascular disease management programs based on community-based participatory research: protocol for a scoping review
Source: Syst Rev. 2021 Sep 24;10:256. doi: 10.1186/s13643-021-01804-4 (PMC8464120; doi:10.1186/s13643-021-01804-4)
Supplement: Supplementary file 1 — Additional file 1:. Appendix. Search Strategies by the Electronic Database [file 13643_2021_1804_MOESM1_ESM.docx]

Appendix. Search Strategies by the Electronic Database

| Database |  | Search terms |
| --- | --- | --- |
| PUBMED | 1 | community-based participatory research[Mesh] |
|  | 2 | community?based participatory research |
|  | 3 | CBPR |
|  | 4 | participatory action research |
|  | 5 | participatory action |
|  | 6 | PAR |
|  | 7 | participat* |
|  | 8 | community engagement* |
|  | 9 | community involvement* |
|  | 10 | civic engagement* |
|  | 11 | civic involvement* |
|  | 12 | 1 OR 2 OR 3 OR 4 OR 5 OR 6 OR 7 OR 8 OR 9 OR 10 OR 11 |
|  | 13 | cardiovascular disease[Mesh] |
|  | 14 | vascular disease[Mesh] |
|  | 15 | cardiovascul* |
|  | 16 | vascular disease |
|  | 17 | vascul* disease |
|  | 18 | 13 OR 14 OR 15 OR 16 OR 17 |
|  | 19 | 12 AND 18 |
| CINAHL | 1 | community based participatory research |
|  | 2 | CBPR |
|  | 3 | 1 OR 2 |
|  | 4 | participatory action research |
|  | 5 | PAR |
|  | 6 | 4 OR 5 |
|  | 7 | participat* |
|  | 8 | 6 AND 7 |
|  | 9 | community engagement or community involvement or civic engagement |
|  | 10 | 3 OR 8 OR 9 |
|  | 11 | cardiovascular disease or cvd or heart or cardiac or coronary heart disease |
|  | 12 | 10 AND 11 |
| Cochrane | 1 | community-based participatory research[Mesh] |
|  | 2 | community?based participatory research |
|  | 3 | community-based participatory research |
|  | 4 | CBPR |
|  | 5 | participatory action research |
|  | 6 | participatory action |
|  | 7 | PAR |
|  | 8 | participat* |
|  | 9 | community engagement* |
|  | 10 | community involvement* |
|  | 11 | civic engagement* |
|  | 12 | civic involvement* |
|  | 13 | 1 OR 2 OR 3 OR 4 OR 5 OR 6 OR 7 OR 8 OR 9 OR 10 OR 11 OR 12 |
|  | 14 | cardiovascular disease[Mesh] |
|  | 15 | cardiovascul* |
|  | 16 | vascular disease[Mesh] |
|  | 17 | vascular disease |
|  | 18 | vascul* disease |
|  | 19 | 14 OR 15 OR 16 OR 17 OR 18 |
|  | 20 | 13 AND 19 |
